# Tropicalization shifts herbivore pressure from seagrass to rocky reef communities

**DOI:** 10.1098/rspb.2022.1744

**Published:** 2023-01-11

**Authors:** Julia Santana-Garcon, Scott Bennett, Núria Marbà, Adriana Vergés, Rohan Arthur, Teresa Alcoverro

**Affiliations:** ^1^ Global Change Research Group, Institut Mediterrani d'Estudis Avançats (IMEDEA), CSIC-UIB, Esporles, Spain; ^2^ Flourishing Oceans Initiative, The Minderoo Foundation, Perth, WA, Australia; ^3^ Institute for Marine and Antarctic Studies, University of Tasmania, Hobart, Tasmania 7001, Australia; ^4^ Evolution & Ecology Research Centre, Centre for Marine Science and Innovation, School of Biological, Earth and Environmental Sciences, University of New South Wales, Sydney, NSW, Australia; ^5^ Nature Conservation Foundation, 3076/5, 4th Cross, Gokulam Park, Mysore, Karnataka 570 002, India; ^6^ Centre d'Estudis Avançats de Blanes (CEAB-CSIC), Accés a la cala Sant Francesc 14, 17300 Blanes, Spain

**Keywords:** global change, temperature, nutrients, exotic species, dietary preference, native and introduced species

## Abstract

Climate-driven species redistributions are reshuffling the composition of marine ecosystems. How these changes alter ecosystem functions, however, remains poorly understood. Here we examine how impacts of herbivory change across a gradient of tropicalization in the Mediterranean Sea, which includes a steep climatic gradient and marked changes in plant nutritional quality and fish herbivore composition. We quantified individual feeding rates and behaviour of 755 fishes of the native *Sarpa salpa*, and non-native *Siganus rivulatus* and *Siganus luridus*. We measured herbivore and benthic assemblage composition across 20 sites along the gradient, spanning 30° of longitude and 8° of latitude. We coupled patterns in behaviour and composition with temperature measurements and nutrient concentrations to assess changes in herbivory under tropicalization. We found a transition in ecological impacts by fish herbivory across the Mediterranean from a predominance of seagrass herbivory in the west to a dominance of macroalgal herbivory in the east. Underlying this shift were changes in both individual feeding behaviour (i.e. food choice) and fish assemblage composition. The shift in feeding selectivity was consistent among temperate and warm-affiliated herbivores. Our findings suggest herbivory can contribute to the increased vulnerability of seaweed communities and reduced vulnerability of seagrass meadows in tropicalized ecosystems.

## 1. Introduction

Climate change is driving a global redistribution of biodiversity with pervasive impacts on ecosystems [1–4]. Disentangling how these changes affect natural systems is a complex challenge owing to the multi-scale, multi-driver and multi-species nature of these changes [5,6]. To date, predictions about how climate change will impact biodiversity largely focus on the direct effects of temperature on individual species, with a particular emphasis on threshold temperatures at which species' range extensions or contractions are likely to occur [7,8]. Less attention has been focused on how warming will modify ecological processes, such as trophic interactions within communities [9–13], which are crucial to ecosystem function and resilience.

Trophic interactions are influenced by animal behaviour and life-history traits which may change under different temperatures. However, these temperature dependencies may not be adequately reflected in the physiological metrics typically used to assess thermal sensitivity, such as critical or lethal temperature limits. For example, in freshwater fishes, preferred temperature and optimal reproductive temperatures are 2–5°C below optimal growth temperatures and 10–15°C below lethal or critical thermal limits [14]. Variation in the thermal range that biological processes depend on is important because it implies that population maintenance (e.g. reproduction and recruitment), or competitive ability, may be impaired well below lethal temperature limits of a species, with flow-on effects for the realized functional impact of a species in a community [14,15]. Therefore, quantifying the temperature sensitivity of specific interactions is essential to understand how ecosystem function may change with warming [16].

Climate-driven effects on species interactions are particularly important in marine systems, many of which are strongly regulated by top-down forces [17]. Marine herbivores, for instance, can have a profound effect on the abundance of benthic primary producers [18] and can play a key role in the resilience of benthic ecosystems [19]. Changes in plant–herbivore interactions, especially on foundation species, can lead to substantial ecological changes and even regime shifts [20,21]. Plants and ectothermic animals, such as fish and invertebrate herbivores, respond predictably to temperature, characterized by a hump-shaped curve in performance. Physiological performance typically increases with temperature up to an optimal threshold and then declines with further increases in temperature toward a critical thermal limit [22]. If optimal temperatures differ between producers and consumers, there may be a mismatch in performance, whereby the performance of warmer-affiliated species continues to increase, while temperate-affiliated species' performance declines, potentially exacerbating or dampening the effects of temperature on plant–herbivore interactions [9,16]. This is particularly relevant for range-shifting species, whose optimal temperatures may be considerably higher than the cooler-affiliated species they encounter as they move poleward. Ocean warming is therefore expected to favour range-extending herbivores and potentially exacerbate impacts on producers and competition for resources with existing cooler-affiliated herbivores.

Despite increasing experimental evidence of the effects of temperature on herbivory [23,24], how these processes manifest in natural systems remains poorly understood. To date, studies of the effects of herbivory in natural marine systems have primarily focused on the population-level impacts, where population size of both primary producers and consumers can have an important effect on realized herbivore impacts [19]. Currently, it remains unclear whether the individual rates of herbivory and the processes that regulate population size display similar thermal responses or not—which has important implications for how functional impacts change with temperature.

In addition to temperature, local and regional variation in nutrient conditions can have a strong impact on patterns of herbivory and in turn benthic habitat structure. Herbivores can display strong dietary preferences in accordance with the nutritional values of marine plants [25,26]. Moreover, low nutrient conditions can lead to compensatory effects on feeding (i.e. higher grazing rates under low nutrient conditions to meet metabolic demands) and reduce primary production, leading to greater vulnerability to overgrazing [27]. This is important in the context of range-shifting herbivores, many of which have a generalist diet, being capable of feeding on multiple species of both seagrass and seaweed [28,29]. Such dietary plasticity raises the prospect that changes in nutrient conditions could alter the feeding choices of species, influencing the realized functional impacts of herbivores on benthic communities. Unpacking how broad-scale changes in temperature and nutrient regimes influence plant–herbivore interactions is therefore critical to understanding the effects of climate change on coastal marine ecosystems.

The Mediterranean Sea provides a particularly interesting region to examine the effects of temperature, nutrients and climate change on herbivory. The Mediterranean is warming two to three times faster than the global ocean [30] and displays a strong longitudinal gradient in temperature, with warmer temperatures in the east and cooler temperatures in the west. In addition, lower nutrients and higher salinity levels are observed in the east compared with the west, and coastal waters have become increasingly oligotrophic over the past decade in response to warming [31]. Species composition of the Mediterranean Sea is also changing in line with climate change expectations, largely as a result of tropical species entering from the Red Sea through the Suez Canal (i.e. known as Lessepsian species, like *Siganus luridus* and *Siganus rivulatus*; [32]). As waters warm, these species are moving westward and establishing in native temperate-affiliated communities [33–35]. Collectively, patterns of temperature, nutrients and species composition from the western to eastern Mediterranean represent a gradient in tropicalization, analogous to observed latitudinal patterns of tropicalization along eastern continental margins (see examples in [20]), and provide a space-for-time substitution that may facilitate insights into the trajectory of coastal marine ecosystems in response to climate change [36].

Changes in herbivory in response to tropicalization are particularly relevant in the Mediterranean where rocky reefs and seagrass meadows form intermixed habitat mosaics over which fish assemblages roam. The concentration of feeding on one habitat or another, therefore, can have a fundamental impact on these respective communities whereby preference for one may indirectly provide protection for the other. Currently, temperate-affiliated herbivores are known to feed on both seagrass meadows and algal communities in the western Mediterranean [37,38], while introduced tropical herbivores in the eastern Mediterranean, are reported to have profoundly transformed rocky reef communities, turning algal forests into barren regions in some areas [39–41]. How changes in temperature and nutrient regimes might influence these dynamics remains an open question, with important implications for the resilience of coastal marine ecosystems to climate change.

Here we examine how the impacts of fish herbivory change across the Mediterranean Sea, along a steep gradient of tropicalization. Specifically, we ask: (1) How do the relative abundance and biomass of fish herbivores change across the Mediterranean Sea? (2) Do *per capita* feeding rates and feeding selectivity by herbivores change in response to temperature and nutrient regimes across the Mediterranean Sea? (3) Do patterns of herbivory differ between temperate-affiliated native species and warm-affiliated exotic herbivores? Finally, by combining relative abundance and biomass of fishes with *per capita* feeding patterns, we compare the gross impact by fish herbivores on seagrass and macroalgae-dominated reef habitats across the Mediterranean.

## 2. Methods

### (a) Study area

**Figure 1.**
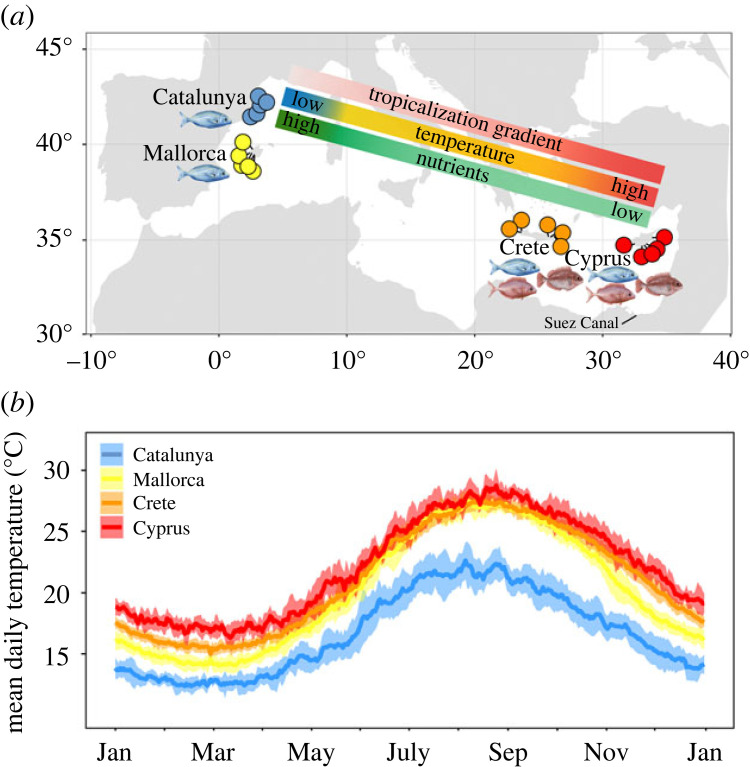
(*a*) Map of tropicalization gradient and study sites within each location (shown as coloured points), spanning 8° of latitude and 30° of longitude across the Mediterranean Sea. (*b*) Daily temperature profiles in Catalunya, Mallorca, Crete and Cyprus. Temperatures represent mean daily temperatures averaged between years over the period 2010–2019. Shaded areas represent the standard deviation in daily temperatures between years.

We sampled at four locations of the gradient of tropicalization in the Mediterranean Sea: Catalunya and Mallorca in the western Mediterranean basin, and Crete and Cyprus in the eastern tropicalized basin (figure 1*a*). The study area spans 8° in latitude and 30° in longitude between Catalunya and Cyprus. Five shallow sites with similar habitat were selected and sampled at each location. Sites ranged between 1 and 8 m in depth and each represented mixed-habitat mosaics of *Posidonia oceanica* meadows and shallow rocky reefs dominated by turf and macroalgal beds. All sampling was conducted between late spring and summer (i.e. June to September in 2017 and 2018) to avoid seasonal effects.

### (b) Sea temperature and nutrient measurements

Underwater temperature loggers (ONSET Hobo pro v2 Data logger) were deployed at collection sites in Catalunya, Mallorca, Crete and Cyprus and left to record hourly temperatures for 12–18 months between 2016 and 2019. In order to extend the temperature record, we used satellite images of sea surface temperature (SST). In particular, we used daily SST maps with a spatial resolution of 0.25°, obtained from the National Centre for Environmental Information (NCEI, https://www.ncdc.noaa.gov/oisst [42]). These maps were generated through the optimal interpolation of Advanced Very High-Resolution Radiometer (AVHRR) data for the period 1981–2019. The closest points to the collection sites were extracted from the satellite images and the resulting time series calibrated against the *in situ* data. Corrected SST data were used to reconstruct daily habitat temperatures from 1981 to 2019.

Total nitrogen and carbon content were measured on leaves of entire shoots of seagrass, *P. oceanica*, and in macroalgal samples of *Padina pavonica* thalli and scraped turf algae. These species were selected as they were dominant at all sites across the study and were commonly consumed by herbivores. *Posidonia oceanica* samples (*n* = 10 shoots per location) were collected from all four locations, while turf and *P. pavonica* samples (*n* = 10 per species and location) were only collected from Catalunya, Mallorca and Cyprus owing to logistical constraints during sampling. Samples were cleaned to remove conspicuous epiphytes and samples were dried for 48 h at 60°C, ground and analysed at Unidade de Técnicas Instrumentais de Análise (University of Coruña, Spain) with an elemental analyser FlashEA112 (ThermoFinnigan).

### (c) Fish density and biomass

Density and body size of the dominant herbivore fish species (i.e. the native sparid *Sarpa salpa* and the exotic rabbitfish *Siganus luridus* and *Siganus rivulatus*) were surveyed at five sites per location, along twelve 25 × 5 m belt transects (i.e. 125 m^2^) per site. Surveys were conducted using diver-operated stereo–video systems (stereo-DOVs). Stereo-DOVs use video cameras to record transects, allowing the identification, abundance, highly accurate estimates of body size and sampling area to be recorded [43]. Stereo-DOVs have proven to be a robust, standardized technique to survey shallow fish assemblages [19,44,45], including in the Mediterranean Sea [46]. The software ‘EventMeasure Stereo’ (www.seagis.com.au) was used for video analysis following the guidelines described in Goetze *et al.* [43].

Fish biomass (B, in grams per 125 m^2^ transect) was calculated from the density (i.e. number of fish per 125 m^2^ transect) and total length (TL) data using length–weight relationship equations for each species (*B* = *a*TL*^b^*). Length–weight parameters were obtained from FishBase using only reference studies from the Mediterranean Sea [47]. Mouth area (MA, in cm^2^) was also estimated using an allometric model (MA = *a*TL*^b^*) that defines the relationship between the fish total length and the mouth area for the species *S. salpa* [48], where *a* is the coefficient in shape (*a* = 0.0005) and *b* is the power fulfilling the dimensional balance (*b* = 2.4146). Mouth-allometry information was not available for the two *Siganus* species assessed, and therefore *S. salpa* allometry was also used for these species, given their morphological similarity.

### (d) Benthic composition

Benthic composition was quantified from the same stereo-DOV transects as the fish density [49], using the same sample design (i.e. five sites per location and twelve 25 × 5 m belt transects per site). For each transect, five frames were extracted across the transect, approximately every 5 m (i.e. 20 s of video). For each frame, three predetermined fixed points in the screen were used to identify the benthos using the following categories: (1) *P. oceanica*, (2) turf algae (i.e. epilithic algal matrix, EAM; less than 1.5 cm in height), (3) erect macroalgae (1.5–15 cm in height), (4) canopy macroalgae (greater than 15 cm in height), (5) *Cymodocea nodosa*, (6) sand, (7) rubble or others. Therefore, benthic cover was determined at each site by 180 points (i.e. 900 points per location). To facilitate comparison between seagrass and macroalgal cover, the two seagrass categories (i.e. *P. oceanica* and *C. nodosa*) were combined in the results as ‘seagrass cover’ and the three macroalgae categories (i.e. turf, erect macroalgae and canopy macroalgae) are referred to in the text as ‘macroalgal cover’.

### (e) Herbivory measurements

*Per capita* consumption rates (bites per minute) by the three herbivore fish species were quantified following the method described by Fox & Bellwood [50]. Individual fish were actively followed for up to 5 min to record their behaviour, bite rates and the substrate type that was grazed. Observations were discontinued if fish showed an obvious response to the presence of the diver and were excluded if they lasted under 2 min. To reduce behavioural differences between fish size classes, only individuals larger than 10 cm were assessed, and those between 10 and 30 cm were prioritized. The number of bites per minute taken on different substrates was classified in the field as on either (1) *P. oceanica,* (2) *C. nodosa*, (3) turf algae (i.e. EAM, less than 1.5 cm in height), (4) erect macroalgae (1.5–15 cm in height), (5) canopy macroalgae (greater than 15 cm in height) or others. Substrate types were grouped for analysis as seagrass (i.e. almost exclusively *P. oceanica* but occasionally *C. nodosa*) and macroalgae (i.e. mostly turf algae and erect macroalgae dominated by *P. pavonica* among others). Habitat was standardized across all observations so that the fish that were followed had the option to feed on macroalgae and seagrass *P. oceanica* meadows within any given observation.

Feeding selectivity between habitats was quantified using Ivlev's electivity index to standardize bite rates based on habitat availability [51]. Ivlev's index is calculated as *E_i_* *=* (*r_i_* − *p_i_*)/(*r_i_* *+* *p_i_*), where *r_i_* is the proportion of substrate *i* in the diet and *p_i_* is the proportion of substrate *i* in the habitat. Values range from −1 to 1, where −1 represents strong selection against a substrate type and 1 represents strong selection for a substrate type. Other variables recorded per individual observation included depth, temperature, time of the day, fish size (in 10 cm size classes) and school size (i.e. the number of fish in the school where the fish being followed spent most time; electronic supplementary material, table S1). Observations were restricted to daylight hours, avoiding dawn and dusk, and were done snorkelling to maximize the time spent in the water (depth restricted between 1 and 5 m).

Mean bite density (i.e. number of bites per minute and per 125 m^2^ transect) for each study location was estimated by combining the mean *per capita* consumption rates and fish density estimates for each species and site. In order to take into account differences in consumption impact resulting from fish of different sizes, consumption pressure was estimated by combining mouth area estimates (MA) and mean bite density. Thus, consumption pressure at each location is defined as the number of cm^2^ that are consumed per minute and per 125 m^2^ of habitat.

### (f) Statistical analysis

Winter and summer warming rates were each analysed using linear regression of the 1st and 99th percentile daily temperature, respectively, recorded each year between 1981 and 2019 in the four locations. Quantile regression spline models were applied to analyse the relationship between individual bite rates per minute and environmental temperature. Feeding rate observations are inherently variable, owing to the range of behaviours that individuals display over a given 5 min observation. As such, low or zero feeding rates do not necessarily imply physiological limitation, but may reflect behavioural variation (e.g. swimming, schooling, resting) during a particular observation. Maximum feeding rates, by contrast, may be informative about the upper limits of fishes' feeding potential at a particular temperature. Quantile regression spline models accommodate this variation by capturing the upper bounds of feeding rates a species exhibits across a range of temperatures [52,53]. We fitted spline models to the 95th percentile of bite rates using the 'rq()' function, as part of the ‘quantreg’ package in the R computing software (v. 4.0.2, R Development Core Team 2020). The ‘bs()’ function as part of the ‘spline’ package was used to construct B-spline basis expansions to fit a piecewise polynomial of specified degree. The most appropriate degree for the polynomial was assessed among a set of models of degree = 1, 2, 3 and 4 for each species using the sample-corrected version of Akaike's information criterion (AIC). The model with the lowest AIC value out of the set of models having a polynomial degree = 1, 2 or 3 was chosen. Where AIC values of multiple models were within 2 units of each other, the model with best visual fit was selected (following [52]).

Patterns in mean fish density, biomass and consumption pressure were each analysed using analysis of variance (ANOVA). For fish, relative abundance and biomass were pooled between transects and standardized per 125 m^2^ within each site (*n* = 5 sites per location). Biomass estimates were log-transformed to improve normality and homogeneity of variance. Consumption pressure was measured for each survey site (*n* = 5 sites per location). For overall patterns, abundance, biomass and consumption pressure were pooled between species and compared between locations (fixed factor).

Patterns in benthic cover and nutritional quality between macrophyte species and locations were each analysed using two-way ANOVA. For benthic data, values of benthic cover for each habitat category were tallied within each site and compared between locations (*n* = 5 sites per location). For nutritional quality, C : N ratios were compared between *n* = 10 replicate samples for each species and location, with the exception of Crete, where only *P. oceanica* was analysed. The assumption of normality was tested using the Shapiro–Wilks test and checking *Q–Q* plots, and homogeneity of variances was checked with Levene's test and residuals plot. Tukey-HSD analysis was used to examine differences between groups for significant main effects.

## 3. Results

### (a) Temperature regimes

A steep east–west gradient in temperature was observed across the four locations, with the coolest temperatures recorded in Catalunya and warmest conditions in Cyprus (figure 1*b*). Annual temperatures at experimental sites ranged from 11 ± 0.3 to 24.5 ± 0.3°C in Catalunya, 13.4 ± 0.2 to 28.2 ± 0.2°C in Mallorca, 14.7 ± 0.1 to 28.4 ± 0.2°C in Crete and 15.1 ± 0.3 to 29.6 ± 0.4°C in Cyprus. Temperature differences between locations have increased over the past 40 years owing to faster warming rates in the east than the west. Minimum temperatures have warmed by 0.23, 0.2, 0.49 and 0.57°C decade^−1^ in Catalunya, Mallorca, Crete and Cyprus, respectively, since 1981 (electronic supplementary material, figure S1). Maximum temperatures have warmed by 0.1, 0.3, 0.6 and 0.4°C decade^−1^ in Catalunya, Mallorca, Crete and Cyprus since 1981 (electronic supplementary material, figure S1).

### (b) Herbivore biomass and density

**Figure 2.**
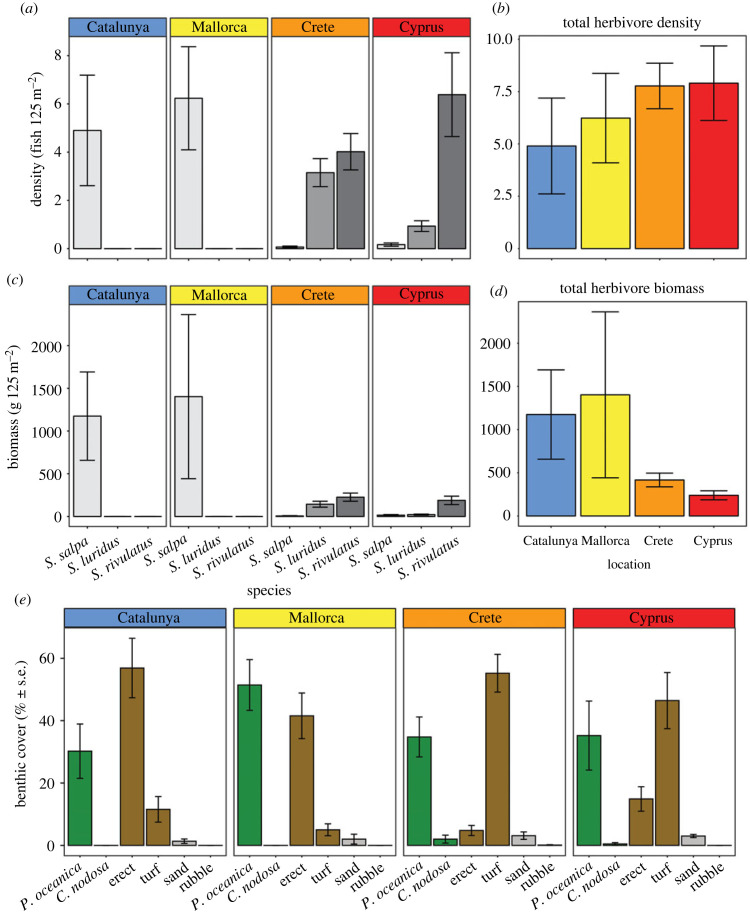
(*a*,*b*) Relative density (average number of individuals per 125 m^2^ transect ± s.e.), (*c*,*d*) biomass (average biomass in grams per 125 m^2^ transect ± s.e.) of fish herbivores and (*e*) benthic cover of seagrass (*Posidonia oceanica*, *Cymodocea nodosa*), macroalgae (turf, erect), sand or rubble (average percentage cover per transect ± s.e.) of sites within four locations across the Mediterranean Sea. Right panels in (*a*) and (*b*) show the aggregated density and biomass of fish species combined per location.

Herbivore density and biomass of the three target species, *S. salpa, S. luridus* and *S. rivulatus*, displayed contrasting patterns across the Mediterranean, with a clear shift in species composition from west to east (figure 2). Overall, herbivore density increased from 4.9 ± 2.3 individuals per 125 m^2^ in Catalunya to 7.9 ± 1.8 individuals per 125 m^2^ in Cyprus, but densities across locations were not significantly different (ANOVA, *F*_3,16_ = 0.497, *p* = 0.69; figure 2*b*). By contrast, overall herbivore biomass decreased from 1174.4 ± 516.8 g per 125 m^2^ in Catalunya to 239.2 ± 52.2 g per 125 m^2^ in Cyprus, but again differences were not statistically significant (ANOVA, *F*_3,16_ = 2.006, *p* = 0.154; figure 2*d*). *Sarpa salpa* significantly declined from west to east (ANOVA, *F*_3,16_ = 7.944, *p* = 0.002) from 100% of total herbivore density and biomass in Catalunya and Mallorca to less than 1% of density and 1.4% of biomass in Crete and 2.1% of density and 6.3% of biomass in Cyprus. High relative abundance of *S. rivulatus* and *S. luridus* compensated for decreased *S. salpa* density in Crete and Cyprus (figure 2*a*), but their twofold smaller size resulted in an overall decrease in herbivore biomass in the eastern Mediterranean (figure 2*b*). The herbivore *Sparisoma cretense* was also present in Crete (0.5 ± 0.14 individual per 125 m^2^) and Cyprus (0.4 ± 0.1 individual per 125 m^2^), but the low abundances meant it was excluded from further analyses in this study.

### (c) Benthic composition

Benthic composition in all selected 20 sites across the Mediterranean was constituted of mixed habitat mosaics of seagrass meadows and algal-covered rocky reef (figure 2*e*). Seagrass cover was dominated by *P. oceanica* and displayed consistent cover between locations (ANOVA, *F*_3,16_ = 1.07, *p* = 0.388), with 30 ± 8.7% cover in Catalunya, 51 ± 8% in Mallorca, 36 ± 7% in Crete and 35 ± 11% in Cyprus. Combined algal cover constituted the highest benthic cover in sites and did not differ between locations (ANOVA, *F*_3,16_ = 1.04, *p* = 0.402), representing 68 ± 8% cover in Catalunya, 46 ± 9% in Mallorca, 60 ± 6% in Crete and 61 ± 11% in Cyprus. Overall, combined algal cover was higher than combined seagrass cover (ANOVA, *F*_1,35_ = 9.83, *p* = 0.003). Algal composition in the western basin was predominantly erect species (e.g. *Padina* spp., *Dictyota* sp., *Halopteris* sp.; thallus height between 1.5 and 15 cm) and constituted over 40% of total cover in Catalunya and Mallorca (figure 2*e*). By contrast, turf algae (less than 1.5 cm) were the dominant algal cover in the eastern Mediterranean, where they accounted for approximately 50% of total cover (figure 2*e*).

### (d) *Per capita* feeding response to temperature

**Figure 3.**
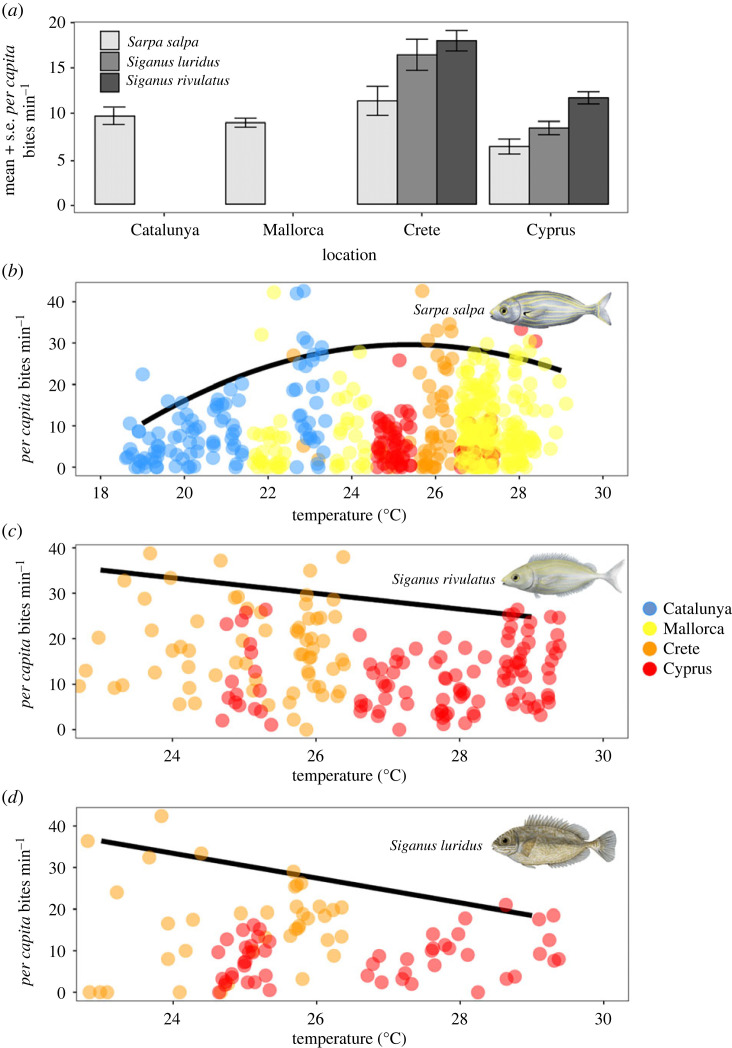
*Per capita* bite rates by fish herbivore species in relation to location (*a*) and *in-situ* habitat temperatures (*b*,*c*,*d*) across the Mediterranean. Bite rates are pooled among seagrass and seaweed food sources. The colour of the points shows the location where the observation was taken. Bite rates were recorded during summer between June and September between 2017 and 2019. Lines represent best-fit 95th quantile regression of bite rates. Bite rates by *Sarpa salpa* were best explained by a second-order polynomial (AIC = 4088, *p* = 0.03), whereas *Siganus* *rivulatus* (AIC = 1361.5, *p* = 0.038) and *Siganus luridus* (AIC = 721.14, *p* = 0.002) were best explained by a first-order model.

*Per capita* feeding rates were recorded for 755 individuals spanning all four locations for *S. salpa* (*n* = 492) and only Crete and Cyprus for *S. luridus* (*n* = 90) and *S. rivulatus* (*n* = 173, electronic supplementary material, table S1), as these species were completely absent in the western locations. For *S. salpa*, there was a significant difference in feeding rates between locations (ANOVA, *F*_3,473_ = 4.227, *p* = 0.005). *Sarpa salpa per capita* feeding rates were significantly lower in Cyprus (6.37 ± 0.83 bites min^−1^) than in Catalunya (9.6 ± 0.9) and Crete (11.3 ± 1.6 bites min^−1^), when all dietary choices were combined (Tukey HSD *p* < 0.05, figure 3*a*). *Per capita* feeding rates were significantly higher in Crete than Cyprus for both *S. rivulatus* (ANOVA, *F*_3,160_ = 29.83, *p* < 0.001) and *S. luridus* (ANOVA, *F*_3,76_ = 24.31, *p* < 0.001, figure 3*a*).

When compared across temperatures*,* feeding rates were variable and displayed non-significant patterns in mean values across a range of 11°C for *S. salpa* (19–29°C, *F*_1,9_ = 3.27, *p* = 0.104, *R*^2^ = 0.18) and 7°C for *Siganus* spp. (23–29°C, *F*_1,5_ = 2.82, *p* = 0.154, *R*^2^ = 0.23). Maximum *per capita* bite rates of *S. salpa* were best explained by a second-order polynomial relationship to temperature (degree = 2, AIC = 4088.9, *p* = 0.032, figure 3*b*). Maximum *per capita* feeding rates showed a subtle linear decline in the 23–29°C range for both *S. rivulatus* (degree = 1, AIC = 1361.5, *p* = 0.038, figure 3*c*) and *S. luridus* (degree = 1, AIC = 721.14, *p* = 0.002, figure 3*d*).

### (e) Feeding selectivity and plant nutritional quality

**Figure 4.**
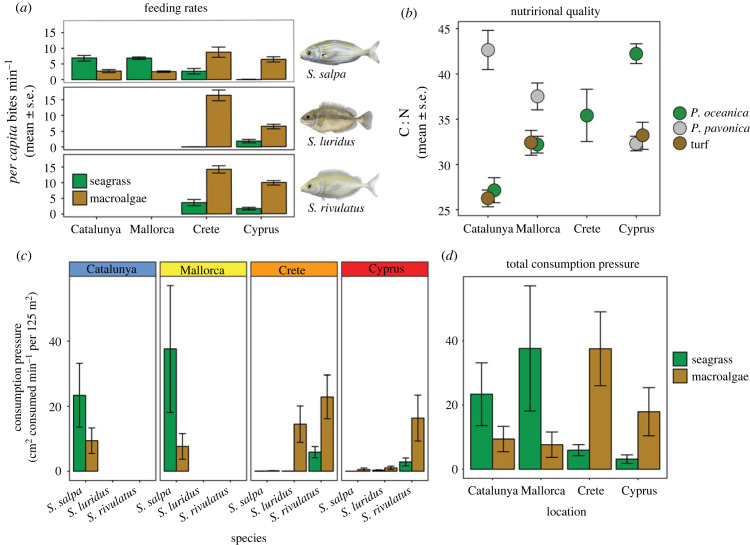
Patterns of feeding selectivity by fishes (*a*), nutritional quality of substrate types (*b*) and consumption pressure by fish herbivores (*c*,*d*) across the Mediterranean Sea. Feeding selectivity represents mean *per capita* bite rates ± s.e. on seagrass (primarily *Posidonia oceanica*) and combined macroalgae (turf, erect and canopy algae). Nutritional quality illustrates mean ± s.e. C : N ratios of plant material in each location. *Padina oceanica* was used to measure seagrass and *Padina pavonica* was used to represent erect macroalgae nutrient content, as these were the two dominant species observed within these functional groups across the study. Mean (±s.e.) consumption pressure represents the combined outcome of abundance, individual gape size and bite rates by fish species (*c*) or herbivore assemblage (*d*) on seagrass meadows and macroalgal beds in Catalunya, Mallorca, Crete and Cyprus.

Feeding rates of herbivorous fishes displayed a significant interaction between location and substrate type (ANOVA, *F*_3,976_ = 86.89, *p* < 0.001), characterized by high to low rates on seagrass from west to east and the opposite pattern on macroalgae, i.e. low to high rates from west to east (figure 4*a*). In Catalunya and Mallorca *S. salpa* were observed feeding on seagrass, *Posidonia oceanica* (7.31 ± 0.42 bites min^−1^), at significantly higher rates than on macroalgae (1.86 ± 0.18 bites min^−1^) (Tukey HSD, *p* < 0.001, figure 4*a*). By contrast, in Crete and Cyprus *S. salpa, S. rivulatus* and *S. luridus* all fed at significantly higher rates on algae than on seagrass (figure 4*a*). When feeding patterns were standardized by habitat availability in each site, the same selectivity pattern was observed (electronic supplementary material, figure S2). *Sarpa salpa* displayed higher Ivlev's selectivity index values for seagrass over algae in Catalunya and Mallorca, and all species displayed higher selectivity for algae than seagrass in Crete and Cyprus (electronic supplementary material, figure S2). The change in feeding selectivity from east to west corresponded to a switch in the nutritional quality of seagrass and macroalgae. C : N ratios of *P. oceanica* significantly increased between Catalunya (27.1 ± 1.3) and Cyprus (42.2 ± 1.1), reflecting a decrease in nutritional quality (figure 4*b*, ANOVA, *F*_3,104_ = 21.12, *p* < 0.001). C : N ratios of turf habitat did not differ from *P. oceanica* in Catalunya (26.2 ± 0.9) or Mallorca (32.4 ± 1.4) but were significantly lower (i.e. higher quality) than for *P. oceanica* in Cyprus (33.2 ± 1.5, figure 4*b*, Tukey HSD, *p* = 0.017). C : N ratios of *P. pavonica* decreased from Catalunya to Cyprus, switching from significantly lower nutritional quality than *P. oceanica* in Catalunya (Tukey HSD, *p* < 0.001) to significantly higher quality in Cyprus (Tukey HSD, *p* = 0.005).

### (f) Consumption pressure by herbivores

**Figure 5.**
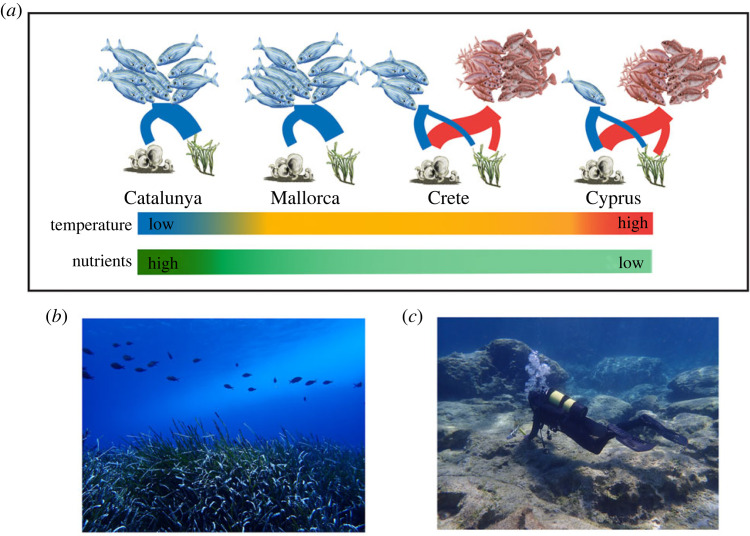
Illustrative summary of the main fish herbivory patterns across the Mediterranean Sea study sites (*a*). Relative abundance of temperate-affiliated *Sarpa salpa* (blue) is high in Catalunya and Mallorca and decreases eastward to Cyprus. Tropical rabbitfish *Siganus rivulatus* and *Siganus luridus* (pink) are not present in Catalunya and Mallorca but are highly abundant in both Crete and Cyprus. Feeding impact by fishes on seaweed and seagrass habitats is depicted by the thickness of connecting lines. Relative magnitude of feeding on seagrass versus algae, feeding rates and population density are illustrated by differences in line thickness. Temperatures are cool in Catalunya, similar between Mallorca and Crete, and highest in Cyprus. Nutritional quality of *Posidonia* was highest in Catalunya and decreased toward the east in Mallorca, Crete and Cyprus. (*c,d*) Photos show contrast between typical seagrass meadows observed across the Mediterranean (*c*) and a typical overgrazed eastern Mediterranean rocky reef habitat (*d*). Photos by A. Verges and S. Bennett.

When *per capita* feeding rates were standardized by relative abundance and bite size of fishes, the overall consumption pressure of herbivores on benthic habitats did not differ significantly between locations across the Mediterranean (*p* > 0.05, figure 4*c*). However, when feeding habitat was considered, two clear shifts from west to east were observed. First, there was a shift in the herbivore species driving consumption pressure, from *S. salpa* in the west to *Siganus* spp. in the east (figure 4*c*, figure 5*a*). Second, there was a significant substrate–location interaction in consumption pressure, from a predominance of seagrass herbivory in the west to a predominance of macroalgae herbivory in the east (ANOVA, *F*_3,32_ = 4.393, *p* < 0.01, figure 5). Consumption pressure on *P. oceanica* was significantly different between locations (ANOVA, *F*_3,16_ = 16.01, *p* < 0.001), characterized by higher pressure in Catalunya and Mallorca than in Crete and Cyprus. Consumption pressure on macroalgae also differed between locations (ANOVA, *F*_3,16_ = 3.413, *p* < 0.043), characterized by lower pressure in the west compared with the east (figure 4*d*).

## 4. Discussion

Disentangling how climate change will impact ecosystems is a complex challenge, owing to multi-scale variability in environmental drivers and ecological processes that interact to shape the structure and function of ecosystems. Here we quantified patterns of fish herbivory on benthic communities across a regional gradient of tropicalization in the Mediterranean Sea. Temperate-affiliated native herbivores were present throughout the region but declined in abundance from cooler to warmer locations (west to east). Warm-affiliated non-native herbivores were highly abundant in the warm, eastern Mediterranean and absent from the west*.* Individual feeding rates of temperate-affiliated herbivores were relatively constant across the Mediterranean, but displayed a pronounced shift in food choice, from seagrass in the west to macroalgae in the east. Similarly, warm-affiliated herbivores fed predominantly on macroalgae, with very few bites taken on seagrass throughout the eastern Mediterranean. The switch in feeding selectivity between west and east matched changes in the nutritional quality of food sources. Overall, combining changes in herbivore assemblages with *per capita* feeding rates of species resulted in similar gross levels of macrophyte consumption along a gradient of tropicalization, but with a fundamental shift in diet, from seagrass in the west to macroalgal habitats in the east.

### (a) Temperature effects on herbivore assemblages

The transition in herbivore assemblage composition across the Mediterranean was broadly consistent with the gradient in sea temperatures and distance to the Suez Canal, the main entrance of warm-affiliated species in the Mediterranean [54]. The absence of siganids from Mallorca is likely to be primarily the result of geography and isolation (e.g. long distance from Suez Canal), although it should be noted that minimum winter temperatures were also cooler in Mallorca than Crete. Currently, siganids are established as far west as the Strait of Sicily, with rare sightings recorded in southern France [54,55]. The abundance of *S. luridus* has been observed to correlate with minimum sea temperatures, with low abundances observed below winter minimums of 14.5°C [56]. However, in Crete, where siganids are abundant, minimum winter temperatures are currently 14.7°C and have warmed by 0.49°C per decade over the past 30 years and therefore would have resembled current Mallorcan winters (13.4°C) less than three decades ago (electronic supplementary material, figure S1). Siganids have been established in nearby Greek waters since the 1950s [28], suggesting that current winter temperatures in Mallorca would be amenable to survival of *Siganus* spp.

At the other end of the spectrum, the declining abundance of *S. salpa* from west to east is consistent with temperatures being too warm, particularly at its trailing edge in Cyprus. Reproduction and recruitment in *S. salpa* occurs during late autumn–winter [57] and warm edge populations have a narrow phenological window that is linked to minimum winter temperatures [58]. Winter temperatures in Cyprus have warmed by over 2°C in the past four decades, potentially having negative effects on *S. salpa* reproduction, recruitment and population size. Further, metabolic studies have demonstrated high thermal sensitivity of *S. salpa* in temperatures above 26°C [59], suggesting potential physiological limitation of this species to both summer and winter temperatures at its warm range edge.

### (b) Temperature and nutrient effects on feeding

Interestingly, while the abundance of *S. salpa* decreased from west to east across the Mediterranean, *per capita* feeding rates were remarkably stable across a 11°C range in temperatures, with feeding rates remaining relatively stable up to 29°C. Feeding rates at 29°C were measured both in Mallorca and in Cyprus, owing to the occurrence of heatwave conditions in the western Mediterranean in 2018, suggesting low temperature sensitivity of feeding rates, independent of geography [60].

Unlike feeding rates, feeding selectivity did change from west to east in parallel to both the distribution of *Siganus* spp. and nutritional quality of food sources. The influence of plant nutritional quality on selectivity and preference by herbivores has been well established in fertilization experiments and across local gradients [25,26]. Here we show that broad-scale changes in nutritional quality between adjacent habitats coincide with altered patterns of herbivory. If nutritional quality of food sources was indeed responsible for patterns in feeding selectivity, this raises the question whether *Siganus* spp. may switch their diet to seagrass upon establishment in the western Mediterranean. A switch in diet assumes that the gut microbiome of *Siganus* spp. is capable of digesting seagrass tissue [61] and that nutritional differences between seagrasses and macroalgae remain the same under future conditions. This idea is supported by findings from translocation experiments of *P. oceanica* from the western Mediterranean to Cyprus, where high feeding rates by *Siganus* spp*.* were observed on the nutrient-rich seagrass from Catalunya and low rates on transplants from Cyprus [62]. In addition to seagrass nutritional quality *per se*, regional differences in epiphyte cover on seagrasses may also have influenced feeding selectivity from west to east. Previous studies have demonstrated that low epiphyte cover of seagrasses results in a switch of feeding preference from seagrass to macroalgae [63]. Finally, changes in schooling behaviour in *S. salpa* may have contributed to their switch from seagrass to macroalgae. *Sarpa salpa* was always present in low abundance in the east and tended to swim and feed in mixed schools with *Siganus* spp. (J. Santana-Garcon 2021, personal observation). *Sarpa salpa* feeding behaviour could in part reflect a ‘peer pressure’ behavioural response to the dominant presence and selectivity of *Siganus* spp. [64].

### (c) Resilience of seagrasses and macroalgae to herbivory

The high feeding pressure by rabbitfish on macroalgae observed in our study is consistent with previous studies that have observed large impacts of *Siganus* spp. on seaweed assemblages in the eastern Mediterranean [39,41]. Similarly, the high feeding rates of *S*. *salpa* on *P. oceanica* are also consistent with studies that show intense impact by this herbivore in the western Mediterranean [38]. These regional differences in herbivory have important implications for the overall impact of macrophyte consumption on benthic habitats across the Mediterranean Sea. Seagrasses like *P. oceanica* are generally resilient to herbivory on account of having a basal meristem that is largely protected from grazing and belowground biomass and energy reserves that compensate for losses in leaf area [65]. Many macroalgae by contrast have apical meristems that are readily consumed and no energy reserves. These differences have particular importance for canopy-forming macroalgae such as *Cystoseira* spp. which are critical foundation species but highly susceptible to overgrazing [39]. The loss of canopy-forming macroalgae can lead to turf-dominated habitats, as observed dominating rocky habitats of Crete and Cyprus in the current study. Unlike canopy species, turfs can be highly resilient to herbivory [19]. Indeed, high grazing rates on turfs lead to low-standing biomass and reduced sediment loads, which can in turn stimulate productivity and further grazing [66,67].

Regional differences in feeding selectivity and functional differences in habitat resilience to herbivory may therefore be contributing to the stark contrast of dense seagrass meadows alongside denuded rocky reef habitats in Crete and Cyprus. *Posidonia oceanica* meadows and rocky reef habitat are intermixed throughout the Mediterranean, creating a patchwork of habitat within sites, where fish assemblages roam across both rocky reef and seagrass. The concentration of feeding on one habitat or another, therefore, is a decision made by fishes within scales of a few metres and replicated over the seascape. The striking juxtaposition of high-biomass seagrass and low-biomass turf-covered rocky reef habitats in the eastern Mediterranean highlights how fine-scale nuances in species interactions (e.g. herbivory) can have profound effects on ecosystem function.

Our findings highlight that the ecological impacts from climate warming and species redistribution may not necessarily be linear or reflect direct responses to thermal physiology alone. Indirect responses mediated by changes in species interactions can play an important role in determining ecosystem responses to warming. Similar changes to the strength of existing species interactions and the emergence of new interactions with range-shifting species are taking place in many regions [19–21]. Our findings highlight that fundamental ecological processes, like herbivore feeding rates, do not necessarily correspond to population-level metrics of species performance across a regional climate gradient. In addition, subtle shifts in nutritional quality of food sources can modify herbivore behaviour, leading to increased vulnerability of rocky reef habitats and reduced vulnerability of seagrass meadows.

## Data Availability

Data and code are available from the Dryad Digital Repository: https://doi.org/10.5061/dryad.nzs7h44vr [68]. The data are also provided in the electronic supplementary material [69].
